# P27 Challenging management of GI complications in Systemic Sclerosis

**DOI:** 10.1093/rap/rkac067.027

**Published:** 2022-09-28

**Authors:** Sultan Iqbal, Abdul Basit

**Affiliations:** Croydon University Hospital, Croydon, United Kingdom; Croydon University Hospital, Croydon, United Kingdom

## Abstract

**Introduction/Background:**

Systemic sclerosis is a chronic, progressive, multisystemic autoimmune disease in which there is excessive collagen deposition in the dermis and internal organs. It is commonly categorized into limited cutaneous or diffuse cutaneous systemic sclerosis. In more than 80% of patients there is GI involvement. Gut complications can impair oral intake, absorption and even faecal continence, and consequently can have severe adverse effect on quality of life. With disease progression, management of GI symptoms may become more of a challenge. We present a challenging case of SSs with esophageal involvement along with severe regurgitation who eventually responded to a specific prokinetic.

**Description/Method:**

70 y/o female initially presented to gastroenterology clinic in 2013 due to dysphagia to solids and was found to have benign fibrotic oesophageal stricture for which she had therapeutic dilatation, (normal biopsy result). Subsequently, she had barium swallow which showed severe regurgitation. Manometry confirmed severely hypo-contractile oesophagus thus she was referred to rheumatology for suspected connective tissue disorder.

On initial review, she did not describe typical inflammatory symptoms, scleroderma, sclerodactyly, Raynaud’s phenomenon, digital ulcers, calcinosis, telangiectasia or any other connective tissue features but was diagnosed with limited cutaneous systemic sclerosis due to ANA & anti-centromere antibody positivity, severe oesophageal dysmotility and diagnosis of mild pulmonary hypertension (on right heart catheter studies).

Her main disabling SSs symptom was refractory belching, intermittent dysphagia and reflux disease resulting in poor oral intake and resultant malnutrition. She had 4-week trial of antibiotics to treat for presumptive small intestine bacterial overgrowth (SIBO) due to persistent belching but had no evident improvement. She had multiple endoscopies which only showed grade B oesophagitis due to which she remained on high dose PPI (Omeprazole 40mg BD) but showed no recurrent stricture. She was also given dual therapy with Ranitidine 150mg bd but derived no benefit.

In Rheumatology clinic she was started on erythromycin 250 mg BD as a pro-kinetic and in liquid form for better tolerability (due to the dysphagia) with gradual but significant improvement of her upper GI symptoms. Switching liquid form to tablets/capsule form via GP resulted in worsening symptoms so was switched back to liquid form. GI symptoms worsened again in 2017 and impression was tachyphylaxis to erythromycin. She was started on trial of another pro-kinetic (metoclopramide) but symptoms continued worsening. In view of this she was started back on liquid erythromycin and her symptoms gradually improved again and has remained stable since.

**Discussion/Results:**

Systemic sclerosis (SSc) is an incurable connective tissue disorder. It is estimated that approximately 90% of patients with SSc have some form of oesophageal involvement. For first three years, this patient’s main symptoms were her upper GI tract issues with reflux and oesophageal dysmotility causing delayed gastric emptying.

Considering the severity of her GI symptoms, it was of great importance for her to maintain a regular follow up with gastroenterology team for a regular review of symptoms and repeat investigations as needed such as multiple repeat UGI endoscopy to ensure no recurrence of oesophageal stricture, monitoring of GORD, and hydrogen breath testing.

We recommend for patients with severe oesophageal dysmotility to be started on prokinetics early to control GI symptoms and have minimal impact of these on quality of life. Liquid preparation has shown a significant improvement in symptoms in our patient as compared to tablet or capsule form which actually worsened the symptoms.

**Key learning points/Conclusion:**

Dysphagia can be a presenting symptom of systemic sclerosis in some cases. Upper gastro-intestinal symptoms can be severe with significant effect on quality of life.

Management of these symptoms can be challenging and may require a combination of treatments.

Pro-kinetics mainly erythromycin can have a major positive impact in controlling GI symptoms,

Liquid form had better outcome in this case as compared to tablet or capsule form.

Poor control of GI symptoms can result in significant malnutrition which is associated with more aggressive disease progression, so GI symptoms management plays a key role in disease control.

